# The Expanding Therapeutic Potential of Neuronal KCC2

**DOI:** 10.3390/cells9010240

**Published:** 2020-01-17

**Authors:** Bor Luen Tang

**Affiliations:** 1 Department of Biochemistry, Yong Loo Lin School of Medicine, National University of Singapore, Singapore 117596, Singapore; bchtbl@nus.edu.sg; Tel.: +65-6516-1040; 2 NUS Graduate School for Integrative Sciences and Engineering, National University of Singapore, Singapore 119077, Singapore

**Keywords:** GABAergic, Huntington’s disease, K^+^/Cl^−^ cotransporter 2, Rett syndrome, spinal cord injury

## Abstract

Dysfunctions in GABAergic inhibitory neural transmission occur in neuronal injuries and neurological disorders. The potassium–chloride cotransporter 2 (KCC2, SLC12A5) is a key modulator of inhibitory GABAergic inputs in healthy adult neurons, as its chloride (Cl^−^) extruding activity underlies the hyperpolarizing reversal potential for GABA_A_ receptor Cl^−^ currents (E_GABA_). Manipulation of KCC2 levels or activity improve symptoms associated with epilepsy and neuropathy. Recent works have now indicated that pharmacological enhancement of KCC2 function could reactivate dormant relay circuits in an injured mouse’s spinal cord, leading to functional recovery and the attenuation of neuronal abnormality and disease phenotype associated with a mouse model of Rett syndrome (RTT). KCC2 interacts with Huntingtin and is downregulated in Huntington’s disease (HD), which contributed to GABAergic excitation and memory deficits in the R6/2 mouse HD model. Here, these recent advances are highlighted, which attest to KCC2’s growing potential as a therapeutic target for neuropathological conditions resulting from dysfunctional inhibitory input.

## 1. Introduction

Inhibitory neural transmission in the adult nervous system is mediated by γ-aminobutyric acid (GABA) [1] and glycine, with fast synaptic inhibition occurring largely through the ionotropic GABA_A_ receptor (GABA_A_R) [2,3]. As a GABA-gated chloride (Cl^−^) channel, the consequence of GABA_A_R signaling is dependent on intracellular Cl^−^ concentration, which determines the reversal potential for GABA_A_R currents (E_GABA_). The intracellular Cl^−^ concentration in neurons is developmentally regulated, with a relatively high postnatal concentration that drops to a lower value going into adulthood. During early neuronal development, GABA_A_R responses are often depolarizing and excitatory, and this property is important for the facilitation of neuronal proliferation, migration, and synaptic integration [4]. The developmental switch of GABA_A_R transmission towards a hyperpolarizing, inhibitory response is due primarily to changes mediated by neuronal sodium-potassium–chloride cotransporter 1 (NKCC1, mediating Cl^−^ influx) [5] and the potassium–chloride cotransporter 2 (KCC2, mediating Cl^−^ efflux), in particular, an enhanced KCC2 surface expression and function shortly after birth [6]. 

KCC2, encoded by *SLC12A5*, is a member of the solute carrier 12 (SLC12) family [7] that is highly expressed in neurons [8,9]. Its function is vital for postnatal survival, as KCC2 knockout mice die quickly after birth due to severe motor deficits that impair respiration [10]. KCC2 transcript levels could be downregulated by neuronal activity [11,12] and brain-derived growth factor (BDNF)-TrkB signaling [13], and its post-translational functional activity is mainly determined by the level of expression at the plasma membrane. The 12-transmembrane KCC2 protein oligomerises, and its membrane trafficking [14], as well as “diffusion-trapping” [15] at the plasma membrane is modulated by neuronal activity-dependent phosphorylation/dephosphorylation of key serine and threonine residues at its cytoplasmic loops. Phosphorylation of S940 of KCC2 by protein kinase C (PKC) [16], for example, is known to stabilize KCC2 at the cell surface and reduces its internalization, whereas excitatory input through the NMDA receptor dephosphorylate S940 by protein phosphatase 1 (PP1) [12] downregulates KCC2’s Cl^−^ efflux activity. Two threonine residues loacated at KCC2’s cytoplasmic tail, T906 and T1007, are phosphorylated by the With no lysine kinase (WNK)-Ste20-related proline/alanine-rich kinase (SPAK)/Oxidative stress response 1 (OSR1) kinase cascade [17,18,19,20], which appears to inhibit KCC2 activity [18]. Recent work with mice knock-ins of KCC2-T906A/T1007A showed that the non-phosphorylatable double mutations increased basal neuronal Cl^−^ extrusion and limit drug-induced epileptic activity [21]. Both the S940A and the T906A/T1007A knock-ins have long-term abnormalities in terms of social behavior and cognitive function [22]. On the other hand, knock-in mice expressing the homozygous phosphor-mimetic KCC2 mutations T906E/T1007E suffer early postnatal deaths from respiratory arrest [23], while heterozygous mice exhibited altered GABAergic inhibition, greater susceptibility to epileptic seizure, and other neurodevelopmental defects [23,24]. Impairment in KCC2 function thus critically affects neurodevelopment and could potentially contribute to the pathogenicity of neurological disorders.

A major human neuropathological condition known to be associated with KCC2 dysfunction is childhood refractory epilepsy [25]. Also, a decrease in KCC2 activity in the spinal cord dorsal horn neurons contributes to central signaling disinhibition, which is an underlying cause of neuropathic pain [26]. KCC2 is therefore an important therapeutic target for these conditions [27,28]. However, due largely to a lack of KCC2 activity-enhancing compounds with good pharmacokinetics [29], experimental and clinical treatment of GABAergic inhibitory dysfunction is better explored in terms of NKCC1 (SLC12A2) inhibition [30], particularly by the diuretic bumetanide [31]. Bumetanide has been shown to be promising in animal models as well as clinical cases of a number of neurological and neuropathological conditions, including Parkinson’s disease [32,33], autism [34,35], schizophrenia [36], newborn analgesia [37], fragile X syndrome [38,39,40,41], and Down’s syndrome [42]. However, unlike the neuronal specific expression pattern of KCC2, NKCC1 is known more widely expressed in other organs including kidney and the respiratory system of mice [43], and likely plays an important role in the inner ear, as knockout of SLC12A2 in mice resulted in deafness and imbalance [44]. The potential undesirable side effects of long-term systemic administration of NKCC1 antagonists therefore makes a search for better KCC2 agonists desirable.

Several recent reports have now further revealed KCC2’s potential as a therapeutic target beyond that of epileptic seizure and neuropathic pain. These include functional recovery from spinal cord injury [45] and amelioration of disease phenotypes of Rett Syndrome [46]. Importantly, these works are based on reagents that enhance KCC2 levels or activity. For an overview of the therapeutic use of NKCC1 antagonists in neuropathological disorders, the reader is referred to the excellent review by Ben-Ari [31]. In the paragraphs below, the focus is on recent advances that attest to KCC2’s growing potential as a therapeutic target, and KCC2 expression or activity enhancement as a therapeutic strategy.

## 2. KCC2 as a Therapeutic Target in Epilepsy and Neuropathic Pain

One underlying course for epileptic seizures is hyper-excitability due to a loss of critical inhibitory input, or when GABAergic input becomes excitatory instead of inhibitory. The pathogenic role of KCC2 dysfunction in this regard, or the fact that enhanced KCC2 activity could help ameliorate epileptic seizures, have been extensively documented [25,47,48]. Experimentally, pharmacological reduction of KCC2 transport in acute mice brain slices increased the duration of seizure-like events, resulting in continuous clonic-like discharges [49]. Status epilepticus (SE) induced by kainite correlated with dephosphorylation of S940 of KCC2, resulting in internalization of cell surface KCC2 [50]. Mice homozygous for a KCC2-S940A knock-in are viable, have comparable basal levels and activity of KCC2 compared to wild-type mice, and have apparently normal neuronal Cl^−^ extrusion. However, these mice are particularly susceptible to kainite-induced SE that is severe enough to be lethal. Brain slices from these mice also have increased susceptibility to clonic-like discharges [49]. Knock-in mice harboring the KCC2-T906A/T1007A double mutant, which lacks phosphorylation-dependent inactivation, also appeared neurologically normal compared to wild-type mice. However, activity-induced deficits in synaptic inhibition were reduced, which is sufficient to limit epileptiform activity induced by the potassium channel blocker, 4-aminopyridine [21]. Importantly, a range of mutations and variants in *SLC12A5* is now known to confer genetic predispositions to childhood SE [51,52,53,54]. These findings have been reviewed recently by Duy and colleagues [48].

Does pharmacological augmentation of KCC2 levels or activity help refractory seizures in a disease setting? Acute KCC2 downregulation occurs during excitotoxic neuronal injuries, resulting from KCC2 cleavage by the calcium-dependent protease calpain [55,56] and signaling from injury-induced BDNF-TrkB activation [13]. It was recently shown that ANA12, a selective TrkB small-molecule antagonist which crosses the blood–brain barrier efficiently [57], significantly reversed post-ischemic KCC2 downregulation as well as phenobarbital-resistant seizures [58]. Thus, pharmacological reversal of a loss of KCC2 activity could help prevent the development of refractory seizures.

NKCC1 and KCC2 have long been implicated in the development of chronic neuropathic pain following spinal cord injury [59]. Loss of functional expression of KCC2 at the spinal cord dorsal horn neurons is a major contributor to the central disinhibition of GABA and glycine receptor-mediated signaling that characterizes neuropathic pain [26,28]. In this regard, several recent reports have shown the conferment of analgesic effects via different means that either reducing KCC2 downregulation, or upregulating KCC2 function. These interventions include derivatives of the conventional antipsychotics phenothiazine [60], BDNF antagonists [61], suppression of the histone deacetylase HDAC2 by HDAC inhibitors [62], activation of 5-hydroxy-tryptamine (HT)_2A_ receptors [63], as well as alternative medical treatments like electro-acupuncture [64]. As discussed below, other recent advances have also implicated several compounds with similar KCC2 enhancement or agonistic properties with promises in neuropathological applications [45,46]. 

## 3. KCC2 as a Potential Therapeutic Target in Spinal Cord Injury and Rett Syndrome

The potential of KCC2 as a therapeutic target for neuropathological conditions has been broadened by recent works that used or developed reagents that enhance KCC2 levels or activity. Two prominent examples of these advances are highlighted below.

### 3.1. KCC2 Activation Promotes Functional Recovery after Spinal Cord Injury

Spinal injuries could lead to devastating permanent paralysis. In fact, many human spinal cord injuries do indeed result in complete paralysis below injury level, despite being anatomically incomplete. In theory, spared connections should eventually promote some degree of functional recovery [65]. That this often fails to occur suggests that the spare circuitries are functionally dormant. Chen and colleagues [45] tackled this potential dormancy in a mouse model of staggered bilateral hemisections (at the thoracic (T) 7 and T10 levels), in which the lumbar spinal cord is severed of all direct brain-derived innervation (all descending axons passing T10 are severed), but with the sparring of potential relay circuits (those axons crossing the midline between T7 and T10 remained intact). The authors screened a series of compounds particularly for their ability to reactivate the spared, but somewhat dormant, spinal connections upon systemic delivery. Amongst these, only CLP290, a carbamate prodrug of the KCC2 agonist CLP257 [66], showed a significant beneficial effect. In CLP290-treated mice, functional recovery assessed by weight-bearing stepping first appeared by 4–5 weeks and became significant from 7 weeks after treatment.

Importantly, CLP290 does not work with mice suffering from complete lesions, and its administration did not affect axonal regrowth. Exogeneous expression of KCC2 with an Adeno-associated virus vector (AAV-KCC2) promoted recovery of stepping to the extent promoted by CLP290. When Cre-dependent, neuronal cell type-specific expression of exogenous KCC2 was performed, only the vesicular GABA transporter (Vgat) promoter-driven Cre that allowed KCC2 expression in inhibitory interneurons showed comparable functional recovery to that promoted by CLP290 treatment. Furthermore, exogeneous KCC2 appears to have exerted its behavioral recovery effect when expressed between and around the staggered lesions (around T5 and T12), with the AAV-KCC2s delivered through the tail vein breaching the compromised blood–brain barrier (BBB) 3 h after lesioning, but not when the AAV-KCC2 is directly injected into the lumbar segments (L2–L5).

What exactly did CLP290 and exogeneous KCC2 expression change or correct at the lesion site in order to promote functional recovery? Using increased c-Fos levels as a proxy to neuronal activity induced by a treadmill walk, the authors found that injured animals exhibited a concentration of elevated c-Fos in the dorsal horn of the spinal cord, possibly as a result of hypersensitivity to peripheral sensory inputs. CLP290 and AAV-KCC2 normalized this c-Fos distribution, ie., reducing the dorsal horn c-Fos concentration and increasing those of the intermediate and ventral spinal cord, as observed for control mice. Although a GABA agonist L-838,417 [67] (which did not promote functional recovery) also reduced c-Fos-positive neurons in the dorsal horn, it did not elevate c-Fos in the intermediate zones and the ventral region. These spinal cord regions are major termination zone of descending inputs and the increase in their neuronal activity by CLP290 and AAV-KCC2 likely reflected an improved response to descending inputs. The increased efficiency of CLP290 and AAV-KCC2-treated injured spinal cord in relaying descending inputs was also shown by enhanced electromyography responses in the transverse abdominal muscle following cortical stimulation. 

Did CLP290 and KCC2 expression help recovery by reducing the excitability of the inhibitory neurons resulting from the lesioning-induced KCC2 downregulation? The authors showed that this is the case with a Designer receptors exclusively activated by designer drugs (DREADD)-based [68] approach. Specific expression of a DREADD construct using AAV9 vectors around the lesion site of Vgat-Cre mice resulted in a similar functional recovery and c-Fos normalization, as observed with CLP290 or KCC2 treatment. The results of Chen and colleagues indicate that heightened excitability of spinal inhibitory interneurons limits the integration of descending inputs into relay circuits upon lesioning, and this can be effectively corrected by increased KCC2 expression or a reagent that upregulates KCC2 levels or activity. Such a reagent could therefore be therapeutically promising in promoting functional recovery after spinal cord injury.

### 3.2. KCC2 Enhancement Alleviate Neurological Symptoms of Rett Syndrome

Rett syndrome (RTT) is a neurodevelopmental disorder caused by mutations in the X-linked Methyl CpG binding protein 2 (MeCP2) coding gene [69,70]. MeCP2 detects and binds to DNA methylation sites and is thought to act primarily as a transcription repressor [71], although it could also activate transcription in some contexts [72]. RTT patients are almost exclusively female and may appear normal at birth but suffer from a pre-puberty onset of a range of neurological defects. These include a stereotypic repetitive hand movement, a loss of purposeful hand skills, spoken language and social communication (autism-like), as well as varying degrees of epileptic seizure, sleep disorders, and respiratory problems [73]. RTT patients do not succumb to neurodegeneration and could survive well into adulthood and would require long-term care. The devastating neurodevelopmental disease has currently no approved treatments beyond symptomatic care and rehabilitation. RTT animal models exhibit clear GABAergic signaling dysfunction [74] and hyperpolarizing GABAergic inhibition, and both RTT patients and RTT model mice brain have reduced KCC2 levels [75,76,77,78].

Tang and colleagues have previously shown that SLC12A5 is a target of MeCP2 repression [77], and that KCC2 levels are reduced in neurons differentiated from RTT patients’ induced pluripotent stem cells (iPSCs) [79]. Furthermore, overexpression of KCC2 in MeCP2-deficient neurons rescued GABA functional deficits, which suggests that KCC2 might be a potential therapeutic target for RTT [77]. In a more recent report, Tang and colleagues described a screening platform where a 2A-luciferase reporter was inserted before the stop codon of the SLC12A5 locus by CRISPR-Cas9 editing in human ES cells, with neurons differentiated from these cells used for high-throughput, unbiased screening of small molecules [46]. From about 900 compounds screened, the authors identified a group of 14 hit compounds that enhance KCC2 expression, which they termed KCC2 expression-enhancing compounds (KEECs). Interestingly, amongst those identified as KEECs are several FDA-approved drugs, including KW-2449, an inhibitor of the FMS-like tyrosine kinase 3 (FLT3) [80], and 6-bromoindirubin-3′-oxime (BIO), an inhibitor of glycogen synthase kinase 3β (GSK3β) [81], as well as resveratrol, a SIRT1 activator [82], and piperine, a transient receptor potential cation channel subfamily V member 1 (TRPV1) agonist [83]. Furthermore, a number of structurally diverse FLT3 kinase inhibitors, as well as TWS-119, a GSK-3β inhibitor which is structurally unrelated to BIO, also exhibited KCC2 transcript and protein expression elevating capacity. In fact, treatment of brain slices with some of the KEECs could also reduce NKCC1 expression, thus causing a significant increase in the KCC2/NKCC1 ratio. This also effectively caused a hyperpolarizing shift in the GABA reversal potential (E_GABA_).

How would the KEECs affect RTT neurons? In MeCP2-null human KCC2 reporter neurons that are isogenic to the reporter cells above, most of the KEECs, including KW-2449, BIO, and resveratrol, also elevated KCC2 reporter activity. Cultured human RTT neurons had E_GABA_ values of about −50 mV, which is significantly more depolarizing compared to the average value of −70 mV in wild-type neurons. KW-2449 and BIO induced a significant hyperpolarizing shift in the E_GABA_ of RTT neurons to values comparable to that in wild-type neurons. Intracellular [Cl^−^] imaging experiments with the Cl^−^ indicator SuperClomeleon showed a significant increase in the chloride extrusion rate in the RTT neurons treated with KW-2449. Treatment with either KW-2449 or BIO also significantly increased the frequency of miniature excitatory postsynaptic currents (mEPSCs) to values equivalent to wild-type control. The RTT neurophysiology phenotype reversal effects of KEECs were loss with both KCC2 silencing and treatment with KCC2 inhibitors, but were not affected by the NKCC1 blocker bumetanide [30]. This indicate that an agonistic enhancement of KCC2, rather than an inhibition of NKCC1, that was effective in reversing the RTT phenotype. The KEECs also altered RTT neurons’ morphological phenotype, including increasing the nuclei size, length, and complexity of neurites, as well as membrane capacitance (which correlates to cell size) to levels comparable to isogenic wild-type controls. Importantly, these KEECs do not seem to significantly affect neuronal electrophysiology and morphology of isogenic MeCP2 wild-type neurons. This point is crucial from a therapeutic perspective as RTT patients are typically mosaics [84] due to X-chromosome inactivation, with a mixture of neurons harboring either wild-type or mutant MeCP2.

To gauge the efficacy of the KEECs in vivo, the authors administered KW-2449 or piperine intraperitoneally into Mecp2 mutant mice and assessed outcome in terms of reversal of respiratory and locomotion deficits. MeCP2 mutant mice showed an increase in breathing pause rate and a decrease in locomotion activity compared to wild-type mice, and these were significantly reversed with KW-2449 and piperine. Given that the KEECs’ efficacy in ameliorating disease-associated respiratory and locomotion phenotypes, these small-molecule KCC2 agonists could therefore be therapeutically useful for RTT, and conceivably also other neurological disorders with etiologies stemming from KCC2 dysregulation.

## 4. Rebalancing the Chloride—NKCC1 versus KCC2 Targeting

The findings discussed above attest to the notion that enhancing KCC2 levels and activities could bring about therapeutic benefits to a range of neuropathological disorders which etiology stems from a loss of inhibitory GABAergic inputs due to KCC2 dysfunction. Attempts to uncover KCC2 activators for experimental therapeutic purposes have not been particularly fruitful [29]. As mentioned above, for correcting depolarizing E_GABA_, more success has been met with an NKCC1 antagonist [31]. In some disease conditions, particularly when NKCC1 levels or activity are upregulated, the use of bumetanide may indeed be more efficacious. An example case is a recent report on Huntington’s disease (HD) [85,86], a monogenic, autosomal-dominant trinucleotide CAG-repeat expansion within exon 1 of the *HTT* gene, resulting in an abnormally long polyglutamine (polyQ) tract of the mutant Huntingtin (mHTT) protein [87,88]. Although midlife onset of the classical HD disease symptoms are manifested as a movement disorder, psychiatric problems and cognitive impairments could precede the motor symptoms [89,90,91]. HTT is a scaffolding protein with many cellular partners [92]. Dargaei and colleagues noted that previous proteomics and interactomics analyses suggested that KCC2 may be associated with HTT [93,94], and that bioinformatics analysis of the unfolded protein response (UPR)-regulated genes in HD have also revealed a reduction in KCC2 transcripts, but an elevation in NKCC1 transcripts [95]. With the hypothesis that KCC2 function is dysregulated in the HD brain, the authors confirmed by co-immunoprecipitation that both wild-type and mHTT interact with KCC2 [85], but not NKCC1, in hippocampal brain lysates prepared from wild-type and an HD transgenic model, the R6/2 mouse [96]. KCC2 transcript and protein levels are both significantly decreased in the hippocampus of R6/2 mice and that of another HD transgenic model, the YAC128 mouse [97], when compared to wild-type. Interestingly, NKCC1 levels are increased in the R6/2 mice, but not the YAC128 mice.

CA1 neurons from the hippocampus of R6/2 mice have a significantly depolarized E_GABA_ compared to wild-type neurons, and similarly, E_GABA_ depolarization is also demonstrated in the cortical regions of the R6/2 mice brain. For R6/2’s CA1 neurons, this depolarize E_GABA_ translates into a significantly higher baseline spiking activity compared to wild-type neurons. Importantly, the E_GABA_ depolarization in R6/2 neurons has apparently converted GABAergic transmission from being inhibitory to excitatory, as GABA application to these neurons elicited a strong increase in spike frequency that was not observed for wild-type neurons [85]. While treatment with the NKCC1 inhibitor bumetanide did not significantly altered E_GABA_ in wild-type CA1 neurons, it significantly hyperpolarized E_GABA_ in neurons from R6/2 mice (which is consistent with the elevated NKCC1 levels noted earlier). To isolate the relative contributions of the changes in KCC2 and NKCC1 levels to E_GABA_, furosemide (which inhibits both NKCC1 and KCC2) were administered together with bumetanide. While E_GABA_ in wild-type neurons depolarized with the co-treatment, E_GABA_ of the R6/2 neurons did not change significantly. The authors surmised that elevated expression of NKCC1 alone could account for the E_GABA_ depolarization in the CA1 neurons in R6/2 mice. Consistent with this notion, bumetanide abolished GABA-induced spiking of R6/2 mouse neurons but did not alter the spontaneous spiking activity of wild-type neurons. The authors also showed that bumetanide administration, either via intraperitoneal injection or micro-osmotic pumps implanted into the lateral ventricle of the brain, enhanced the mice’s performances in T-maze spontaneous alternation tasks [85]. 

In this particular case, NKCC1 inhibition rather than KCC2 elevation/activation was the intervention administered, and this is effective in the R6/2 model with elevated NKCC1. However, it is unclear whether bumetanide would be equally effective for the YAC128 mice model with no NKCC1 elevation. On the other hand, because there is KCC2 reduction in both RTT mice models (and RTT patient brain [78]), it is conceivable that a KCC2-focused intervention, if available, may also produce beneficial effects. In conjunction with the more ubiquitous nature of NKCC1 expression and potential systemic side effects of NKCC1 antagonism, the new findings highlighted in Section 3 above suggest a number of KCC2 expression enhancers or agonists that are worth further therapeutic exploration.

## 5. KCC2 Enhancers and Agonists—Promises and Caveats

As far as direct KCC2 activation is concerned, CLP290 appears to be a promising pharmaceutical agent. It was previously shown that oral CLP290 administration in mice could effectively prevent KCC2 downregulation in the superficial dorsal horn (SDH) of the spinal cord resulting from morphine treatment, and alleviates morphine-induced hyperalgesia [98]. As outlined above, Chen and colleagues have used it to effectively reactivate spinal injury-induced dysfunctional spinal circuit to a more functional state [45]. In another recent report, Lizhnyak and colleagues showed that CLP290 could also restore lost KCC2 levels and improve behavioral function in traumatic brain injury (TBI) [99]. However, in this case, the timing of CLP290 administration is critical, and the compound is not effective when given outside a certain time window. CLP290 is a prodrug synthesized from the lead compound CLP257 with a carbamate protection of its hydroxyl group from glucuronidation, and has an improved pharmacokinetic profile compared to CLP257 [66]. Whether CLP290 directly modifies KCC2 surface expression [66] and activity has been challenged [100,101], but its effectiveness in multiple neuropathological paradigms is nonetheless encouraging.

**Figure 1:**
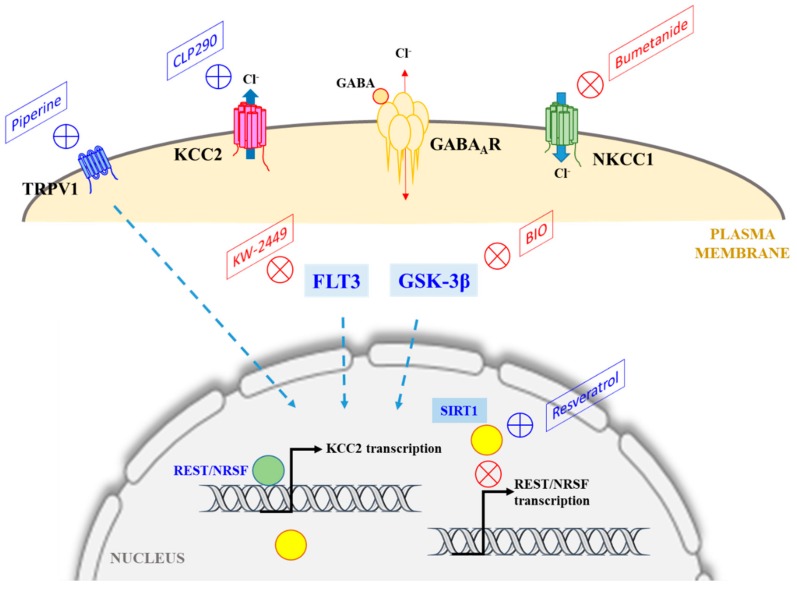
A schematic diagram illustrating the major chloride transporters KCC2 and NKCC1, whose activities determine intracellular Cl^−^ concentration and EGABA, and the pharmacological manipulations that could potentially correct for a loss of KCC2 levels or activity under neuropathological conditions (where GABAergic transmission could become excitatory instead of inhibitory). The classical NKCC1 antagonist bumetanide has been shown to be efficacious in this regard. As discussed in the text, CLP290 enhances the plasma membrane expression of KCC2, while compounds that target TRPV1, FLT3, or GSK-3β also modulates KCC2 expression (dotted arrows), but the mechanisms involved are unclear. Resveratrol activation of SIRT1 could downregulate the expression of RE1-silencing transcription factor/Neuron-restrictive silencer factor (REST/NRSF), which suppresses KCC2 expression. See text for more details.

The finding that several classes of compounds which are FDA-approved drugs targeting various cellular signaling components/pathways could also enhance KCC2 levels and activity are of considerable interest [46] (summarized in Figure 1). From the report of Tang and colleagues [46], it appears that KCC2 expression and activity could be enhanced by inhibiting FLT3 tyrosine kinase and GSK3β, or by activating SIRT1 and TRPV1. How do these compounds and the pathways they affect impinge on KCC2 expression? Would the use of these compounds and the inhibition/activation of their associated pathways be effective in correcting depolarizing E_GABA_ and dysfunctions in GABAergic inhibition? Some further contemplations on these potential KCC2 agonists, particularly with relevance to the major manifestation of GABAergic dysfunctions (epileptic seizure and pain), are made below.

Pathologically speaking, FLT3 is known mainly for its cancer-causing mutations, particularly in hematological malignancies like acute myeloid leukemia [102]. FLT3 is expressed in the brain and neurons [103] and its mutation could also be found in glioma and glioblastoma [104]. The exact mechanism underlying the enhancement of KCC2 expression or activity via FLT3 kinase inhibition by KW-2449 is unclear at the moment. However, it is notable that neuronal FLT3 was recently shown to be involved in neuropathic pain [105]. Immune cells accumulating at nerve injury sites express the Flt3 ligand, and FLT3 activation by intra-sciatic nerve injection of its ligand is sufficient to produce pain hypersensitivity, with changes in gene expression and altered sensitization of sensory neurons. In this regard, FLT3 inhibition may therefore also help to alleviate neuropathic pain. Exploring the mechanism of FLT3 signaling and pain induction in neurons and how FLT3 inhibition may have a wider role in alleviating KCC2 dysfunction-dependent and -independent neuropathological disorders would thus be an interesting and worthwhile pursuit. 

A role for the brain-enriched GSK-3 isoform GSK-3β in neuronal function and various neurological disorders is well-known [106,107,108]. Again, how the GSK-3β inhibitor BIO or TWS-119 might work in the context of enhancing KCC2 expression is unclear. Several other relevant recent reports, however, cautioned against the approach of GSK-3β inhibition in KCC2 dysfunction. It appears that either an increase or a decrease in GSK-3β activity could exacerbate hippocampal damage and increased seizure severity in a model of kainite-induced status epilepticus [109]. Another report has in fact shown that GSK-3β activity alleviates epileptogenesis, possibly via influencing the phosphorylation of the GluA1 subunit of the excitatory AMPA receptor [110]. Furthermore, GSK-3β activity was shown to be enhanced in hippocampus but reduced in the spinal dorsal horn following a model of spare nerve injury, and GSK-3β inhibitors induced persistent pain hypersensitivity in operated animals [111]. Whether the above findings connecting GSK-3β inhibition to neuropathic pain or epileptic seizures are related to KCC2 activity is not known. However, the situation with GSK-3β signaling in neuropathology is likely complex and needs to be examined in more detail before GSK-3β inhibition is deployed as a strategy for any neuropathological disorder. 

The vanilloid receptor TRPV1 is a heat-gated cation channel expressed by sensory neurons and functions mainly in body temperature regulation [112] and nociception [113,114]. TRPV1 antagonists are therefore analgesics for the treatment of various pain conditions [115]. TRPV1 levels increase during epileptic episodes [116], and as there may be a role for calcium ion accumulation through the TRPV1 channel in the etiology of epileptic seizures, TRPV1 inhibition could potentially be beneficial against epileptic onsets [117]. It is again unclear how the TRPV1 agonist piperine enhanced KCC2 levels or activity. However, in view of the above relevant counter indications against TRPV1 in terms of pain and epileptic seizure which accompany neuropathological conditions, therapeutic TRPV1 activation should also be contemplated with caution.

The polyphenolic compound resveratrol is an allosteric activator of SIRT1 [82], and has been extensively implicated in neuroprotection and anti-aging effects [118,119,120]. Its effect on KCC2 expression could potentially be explained. *SLC12A5* transcription is regulated by a repressor element-1 (RE-1) site in its 5′ regulatory region [121], and it has been reported that resveratrol could act via SIRT1 activation to downregulate the expression of RE1-silencing transcription factor/Neuron-restrictive silencer factor (REST/NRSF) [122]. The latter is a downstream target of SIRT1 [123]. Resveratrol is known to be anti-nociceptive and inhibits neuropathic pain [124,125], and also appears to be anti-epileptic [126]. In these regards, its upregulation of KCC2 is in line with a general protection against the consequences of KCC2 dysfunction. Although resveratrol has many targets in the cell and its bioavailability to brain tissues have been in doubt, a recent trial with Alzheimer’s disease patients indicated that resveratrol and its major metabolites do penetrate the BBB and effected CNS biomarker changes [127]. SIRT1 activation by more specific activators like SRT1720 [128] has been shown to alleviate diabetic neuropathic pain [129]. Given the general neuroprotective effect of resveratrol and SIRT1 [130,131], activation of the latter by resveratrol or other more specific activators appears to be a somewhat safer approach to target KCC2 dysfunction. 

In conclusion, multiple pharmacological options, in addition to the NKCC1 antagonist bumetanide, particularly those that enhance either KCC2 levels or activity, which is, in many cases, reduced in neuropathological disorders, have now emerged (see Figure 1). However, much more work is needed to decipher their mechanism of action and possible undesirable side effects in order for them to be therapeutically useful. Importantly, a true KCC2 agonist that would be beneficial would need to upregulate KCC2 in a functional and active manner, which means not just increasing its transcript levels or translated product, but also facilitating proper plasma membrane targeting. The ability of the various pharmaceutical agents to do this should be investigated.
